# Investigation of single and synergic effects of *NLRC5* and *PD-L1* variants on the risk of colorectal cancer

**DOI:** 10.1371/journal.pone.0192385

**Published:** 2018-02-06

**Authors:** Calogerina Catalano, Miguel Inacio da Silva Filho, Christoph Frank, Katerina Jiraskova, Veronika Vymetalkova, Miroslav Levy, Vaclav Liska, Ondrej Vycital, Alessio Naccarati, Ludmila Vodickova, Kari Hemminki, Pavel Vodicka, Alexander N. R. Weber, Asta Försti

**Affiliations:** 1 Division of Molecular Genetic Epidemiology, German Cancer Research Center (DKFZ), Heidelberg, Germany; 2 Department of Molecular Biology of Cancer, Institute of Experimental Medicine of the Czech Academy of Sciences, Prague, Czech Republic; 3 Institute of Biology and Medical Genetics, 1^st^ Medical Faculty, Charles University, Prague, Czech Republic; 4 Department of Surgery, First Medical Faculty, Charles University and Thomayer Hospital, Prague, Czech Republic; 5 Biomedical Center, Faculty of Medicine in Pilsen, Charles University Prague, Pilsen, Czech Republic; 6 Molecular and Genetic Epidemiology, Italian Institute for Genomic Medicine (IIGM), Turin, Italy; 7 Center for Primary Health Care Research, Clinical Research Center, Lund University, Malmö, Sweden; 8 Department of Immunology, Interfaculty Institute for Cell Biology, University of Tübingen, Tübingen, Germany; Istituto di Ricovero e Cura a Carattere Scientifico Centro di Riferimento Oncologico della Basilicata, ITALY

## Abstract

Constitutive activation of interferon signaling pathways has been reported in colorectal cancer (CRC), leading to a strong CD8^+^ T cell response through stimulation of *NLRC5* expression. Primed CD8^+^ T cell expansion, however, may be negatively regulated by *PD-L1* expression. Additionally, aberrant *PD-L1* expression enables cancer cells to escape the immune attack. Our study aimed to select potential regulatory variants in the *NLRC5* and *PD-L1* genes by using several online *in silico* tools, such as UCSC browser, HaploReg, Regulome DB, Gtex Portal, microRNA and transcription factor binding site prediction tools and to investigate their influence on CRC risk in a Czech cohort of 1424 CRC patients and 1114 healthy controls. Logistic regression analysis adjusted for age and gender reported a moderate association between rectal cancer risk and two *NLRC5* SNPs, rs1684575 T>G (OR: 1.60, 95% CI: 1.13–2.27, recessive model) and rs3751710 (OR: 0.70, 95% CI: 0.51–0.96, dominant model). Given that a combination of genetic variants, rather than a single polymorphism, may explain better the genetic etiology of CRC, we studied the interplay between the variants within *NLRC5*, *PD-L1* and the previously genotyped *IFNGR1* and *IFNGR2* variants, to evaluate their involvement in the risk of CRC development. Overall we obtained 18 pair-wise interactions within and between the *NLRC5* ad *PD-L1* genes and 6 more when *IFNGR* variants were added. Thirteen out of the 24 interactions were below the threshold for the FDR calculated and controlled at an arbitrary level q*<0.10. Furthermore, the interaction *IFNGR2* rs1059293 C>T—*NLRC5* rs289747 G>A (P<0.0001) remained statistically significant even after Bonferroni correction. Our data suggest that not only a single genetic variant but also an interaction between two or more variants within genes involved in immune regulation may play important roles in the onset of CRC, providing therefore novel biological information, which could eventually improve CRC risk management but also PD-1-based immunotherapy in CRC.

## Introduction

Colorectal cancer (CRC) is the third most common cancer and the fourth leading cause of cancer mortality worldwide [1]. CRC represents a paradigm for the link between inflammation and cancer [2]. The intestinal tract is continuously exposed to both potential pathogens and beneficial commensal microorganisms; therefore the homeostatic balance between tolerance and immunity represents a regulatory challenge to the mucosal immune system [3]. In this context a pivotal role is played by the epithelial cells that monitor the intestinal microenvironment for pathogenic and commensal microorganisms via so-called pattern recognition receptors (PRRs), e.g. Toll-like receptors (TLRs), and in turn influence the function of antigen presenting cells and lymphocytes [3,4]. Additionally, the gut microbiota provides crucial health benefits to its host by contributing to the regulation of the intestinal immune homeostasis [3, 5]. Recently, it has become obvious that alterations of the regulatory pathways that maintain this homeostasis can result in the development of local and chronic inflammation, inflammatory bowel disease (IBD) and CRC [5]. Aberrant activation of nuclear factor kappa B (Nf-kB) and interferon (IFN) signaling pathways have been reported to play a pivotal role in CRC by triggering the production of several proinflammatory mediators [6–8]. Particularly IFNγ signaling pathway is known to play an important role in controlling the CD8^+^ T cell expansion through the stimulation of *NLRC5* (NOD-like receptor C5) expression, a major histocompatibility complex (MHC) class I transactivator [9,10]. *NLRC5* is a member of the Nod-like receptor (NLR) family of PRR proteins. It contains a nucleotide-binding domain and leucine-rich repeats, which are conserved in PRRs that regulate inflammatory responses and cell death. Given its role in the transcription of MHC class I genes, it is reasonable to think that NLRC5 may play a prominent role in antitumor immunity and its loss may promote tumor immune evasion [11]. Moreover, cytokine production and CD8^+^ T cell expansion is necessary for generating an effective immune defense against invading harmful pathogens [12].

By assuming the importance of a balanced immune response, a physiological feedback mechanism played by PD-L1 (Programmed death-ligand 1) is necessary for terminating the immune responses in a proper way and for maintaining self- tolerance [13]. However, it has recently been shown that IFNγ is also involved in promoting *PD-L1* expression in tumor cells [11]. This results in an aberrant *PD-L1* expression that allows cancer cells to escape the antitumor immune response by suppressing the CD8^+^ T cell expansion [13–15]. This escape mechanism is reversed by immune checkpoint inhibitors targeting the PD-1/PD-L1 interaction and restores anti-tumor immunity [16]. Thus the possibility of PD-1/PD-L1-based therapies has received much attention in many tumor entities including CRC.

To gain further evidence about the potential role of SNPs within *NLRC5* and *PD-L1* genes, we genotyped a set of 16 potential regulatory single nucleotide polymorphisms (SNPs) in a case-control study of 1424 CRC patients and 1114 healthy controls from the Czech Republic and evaluated their association with CRC risk. Moreover, given the opposite actions of these two proteins on the CD8^+^ T cell expansion, we investigated whether pair-wise interactions between all the investigated SNPs and the previously genotyped SNPs in the *IFNGR1* and *IFNGR2* genes exist [17], which may have interactive effects on the risk of CRC. This strategy has the potential to identify complex biological links among cancer-related immunity genes and processes they are involved in, and could provide novel information for a better basic understanding, risk-management and therapy of CRC.

## Materials and methods

### Ethics statement

Written informed consent was given by all participants enrolled in the current research study in accordance with the Helsinki declaration. The project was approved by the ethical committees of the participating institutes, the Institute of Experimental Medicine, Academy of Sciences of the Czech Republic, Prague, Czech Republic (who issued the Institutional Certification for Multicenter Studies on July 16^th^ 2015 covering all studies between 2004–2015) and the Institute for Clinical and Experimental Medicine and Faculty Thomayer Hospital, Prague, Czech Republic (786/09 (G09-04-09) and 622/11(G11-04-09)).

### Study population

The case group contained 1424 CRC patients recruited between the years 2004 and 2013 by several oncological departments in the Czech Republic (Table 1)[17]. Their mean age was 62.7 years, and 61.8% of them were men. The patients showed positive colonoscopic results for malignancy, histologically confirmed as colon or rectal carcinomas. Patients with any previous history of cancer or who met the Amsterdam criteria I or II for hereditary nonpolyposis colorectal cancer were not included in the study. General information about gender and age at diagnosis was available for all patients. The control group contained 1114 healthy individuals recruited by the blood-donor centers in Kralovske Vinohrady Hospital and Vojkov hospital in Prague [17,18]. Their mean age was 47.1 years, and 53.4% of them were men.

**Table 1:** Characteristics of the colorectal cancer patients.

CRC risk analysis		Cases	Controls	p-value
All patients		1424	1114	
Age at diagnosis	Mean (range)	62.7 (24–90)	47.1 (18–94)	**< .0001**^a^
	Median	63	47	
Gender	Male	880 (61.8%)	595 (53.4%)	**2.6e-05**^b^
	Female	544 (38.2%)	519 (46.6%)	
Tumour location	-	-		
	Colon	889 (62.4%)		
	Rectum	398 (27.9%)		
	missing information	137 (9.6%)		

^a^: Z statistics: Wilcoxon Rank-Summ-Test;

^b^: Chi-square.

P < 0.05 are in bold.

### SNP selection

A total of 16 SNPs, which captured 32 potential regulatory SNPs (r^2^ > 0.89), were selected for genotyping within the *NLRC5* (NLR family, CARD domain containing 5) and *CD274* (also known as PD-L1, programmed death ligand 1) genes according to the following selection criteria: non-coding SNPs in the 5’ flanking region (up to 1kb from the transcription start site (TSS) containing the promoter, enhancer or other transcription factor binding sites), 5′ and 3′ untranslated regions (UTRs), and SNPs regulating the expression of the selected genes (eQTL SNPs) with a minor allele frequency (MAF) ≥ 0.10 in the CEU population validated by 1000 Genomes and with a pairwise linkage disequilibrium (LD) r^2^ ≤ 0.80 (S1 Table).

### In-silico analysis

SNPs were selected using several in silico tools, such as UCSC browser (https://genome-euro.ucsc.edu/) to collect all potential functional SNPs in the regulatory regions, HaploReg (http://www.broadinstitute.org/mammals/haploreg/haploreg.php) and Regulome DB (http://www.regulomedb.org/) to explore the chromatin state, conservation, and regulatory motif alterations within sets of genetically linked variants, Gtex Portal (https://gtexportal.org/home/) to identify all cis-eQTL SNPs that affect the expression of genes of our interest and microRNA binding site prediction tools (http://www.microrna.org/microrna/home.do, http://epicenter.iefreiburg.mpg.de/services/microsniper/) to investigate the 3’-UTR and to predict if a SNP within the target site will disrupt/eliminate or enhance/create a microRNA binding site. PERFECTOS-APE (http://opera.autosome.ru/perfectosape/scan) and s-Transcription factor Affinity Prediction (s-TRAP, http://trap.molgen.mpg.de/cgi-bin/trap_two_seq_form.cgi) were used to identify transcription factors whose binding sites can be significantly affected by a given polymorphism. LD and the haplotype blocks within the genes were examined based on r^2^ (S1 Table).

### Genotyping

In this project, genomic DNA from peripheral blood leukocytes was used. The KASP (LGC genomics, Hoddesdon, Hertfordshire, UK) and the TaqMan (Thermo Fisher Scientific, Darmstadt, Germany) allelic discrimination methods were used to genotype the selected SNPs. The genotyping was performed blinded by the case–control status of each sample. DNA amplification was performed according to the LGC genomics’ and TaqMan´s PCR conditions. Genotype detection was performed using ViiA 7 Real-Time PCR System (Thermo Fisher Scientific). The sample set contained 142 duplicated samples as quality controls. The genotype correlation between the duplicate samples was > 90%. Genotype call rate ranged between 94.0 and 100%.

### Statistical analysis

The observed genotype frequencies in the controls were tested for Hardy–Weinberg equilibrium (HWE) using the chi-square test. Odds ratios (ORs) and 95% confidence intervals (CIs) for associations between genotypes and CRC risk were calculated by logistic regression (SAS Version 9.3; SAS Institute, Cary, NC), and adjusted for age and gender. The estimated power was >98% for OR ≥ 1.5 (MAF > 0.10; p = 0.05; dominant model) and >98% for OR ≥ 1.5 (MAF > 0.50; p = 0.05; recessive model) (Quanto: http://hydra.usc.edu/gxe/).

All possible SNP combinations were evaluated in binary interaction to find the SNP-SNP interactions that best predict the disease risk. In addition to the SNPs genotyped in the current study, we also included all SNPs in the *IFNGR1/2* genes genotyped previously in 1327 CRC patients and 758 controls from the same Czech cohort [17]. Four different modes of inheritance were calculated and tested for each pair: the so called “three genotypes model” whereby each SNP was treated as a categorical variable with three levels (genotypes); the “log additive model” whereby SNPs were modeled as a continuous variable and genotypes were converted into 0, 1 or 2 risk alleles; the “dominant model” whereby AA was used as reference and AB and BB as the test group; and “the recessive model” whereby AA and AB were used as reference group and BB as the test group. Likelihood ratio tests were performed to assess whether including the SNP–SNP interaction term led to a significantly better fit of the data. The SNPs that significantly interacted with each other according to several competing models were ranked according to Akaike information criterion (AIC). The smaller the value of AIC, the better the model data fit. To assess the contribution of all genetic components (both SNPs and interaction term) to the model, likelihood ratio test-based P-values were computed. For the best model of each SNP pair, the corresponding ORs and the Wald estimates for their confidence intervals (CIs) and P-values were calculated. Altogether, 120 (16 SNPs*(16–1)/2) independent tests were carried out, leading to a Bonferroni corrected p-value of 0.05/120 = 0.0004. In addition, as an alternative approach, we controlled the false discovery rate (FDR) using the Benjamini-Hochberg procedure. The p-values were sorted from the smallest to the largest and ranked in ascending order. The false discovery rate was calculated and controlled at an arbitrary level q* < 0.10, defining q = mP_(1)_/i, where m is the number of multiple tests, P the p-value of each interaction and i the Rank. Analysis was performed using R version 3.3.2.

## Results

### CRC risk

As shown in Table 1, there was a significant difference in the age and sex distribution between the cases and controls (p-value <0.0001 and p-value 2.6e-05, respectively). The genotype distribution of all 16 genotyped polymorphisms was consistent with HWE in the control group (P > 0.05). Logistic regression analysis adjusted for age and sex reported an association between rectal cancer risk and 2 *NLRC5* SNPs, rs1684575 (OR: 1.60, 95% CI: 1.13–2.27, recessive model) and rs3751710 (OR: 0.70, 95% CI: 0.51–0.96, dominant model) (S2 Table). The other genotyped SNPs did not show any association with CRC risk (S2 Table).

### Possible effect of SNP-SNP interactions on CRC risk

We further investigated whether SNP-SNP interactions among these 16 SNPs within *NLRC5* and *PD-L1* genes could affect colorectal cancer risk. Eighteen interactions, including interactions between SNPs both within a gene and between the two genes, were detected at a significance level of p-value <0.05 (Table 2), however, none of these interaction term p-values survived the conservative Bonferroni multiple testing correction (p-value< 0.0004); although the global null hypothesis test was highly significant (p-value< 0.0001). When we calculated and controlled the FDR at an arbitrary level q* < 0.10, a total of 12 of these interactions were below the given threshold (S3 Table). For the best model of each SNP-SNP interaction, the association with CRC risk was evaluated (S5 Table).

**Table 2:** *NLRC5-PD-L1* pair-wise interactions with cases and controls. Only the best genetic model of each SNP pair is shown.

SNP1	SNP2	Mode of inheritance SNP1	Mode of inheritance SNP2	LRT Statistic	DF	p-value based on LRT	LRT Statistic	DF	p-value based on LRT
				(interaction term)			(SNPs total)		
rs27194	rs289726	Three genotypes	Dominant	13	2	**0.002**	15.54	5	**0.008**
rs289726	rs822338	Dominant	Three genotypes	11.73	2	**0.003**	12.12	5	**0.033**
rs12445252	rs43216	Recessive	Dominant	9.34	1	**0.002**	11.12	3	**0.011**
rs3751710	rs4143815	Three genotypes	Recessive	8.63	2	**0.013**	11.52	5	**0.042**
rs2890657	rs289747	Dominant	Dominant	7.96	1	**0.005**	8.52	3	**0.036**
rs2890657	rs56315364	Recessive	Dominant	7.77	1	**0.005**	8.03	3	**0.045**
rs10815225	rs289726	Recessive	Dominant	7.74	1	**0.005**	11.12	3	**0.011**
rs12445252	rs2890657	Dominant	Recessive	7.66	1	**0.006**	7.93	3	**0.048**
rs27194	rs289748	Recessive	Dominant	7.61	1	**0.006**	8.25	3	**0.041**
rs2890657	rs289748	Recessive	Dominant	7.23	1	**0.007**	8.31	3	**0.04**
rs289747	rs822338	Dominant	Dominant	6.98	1	**0.008**	7.98	3	**0.046**
rs10815225	rs4143815	Recessive	Recessive	6.86	1	**0.009**	11.7	3	**0.009**
rs27194	rs56315364	Recessive	Three genotypes	6.79	2	**0.034**	11.88	5	**0.037**
rs27194	rs4143815	Recessive	Dominant	6.62	1	**0.01**	8.02	3	**0.046**
rs10815225	rs1684575	Recessive	Three genotypes	6.08	2	**0.048**	12.45	5	**0.029**
rs158483	rs866066	Recessive	Recessive	5.66	1	**0.017**	8.04	3	**0.045**
rs27194	rs43216	Recessive	Recessive	4.39	1	**0.036**	8.73	3	**0.033**
rs289748	rs56315364	Recessive	Recessive	4.13	1	**0.042**	10.27	3	**0.016**

DF: Degrees of Freedom

LTR: Likelihood Ratio Test

**Fig 1:**
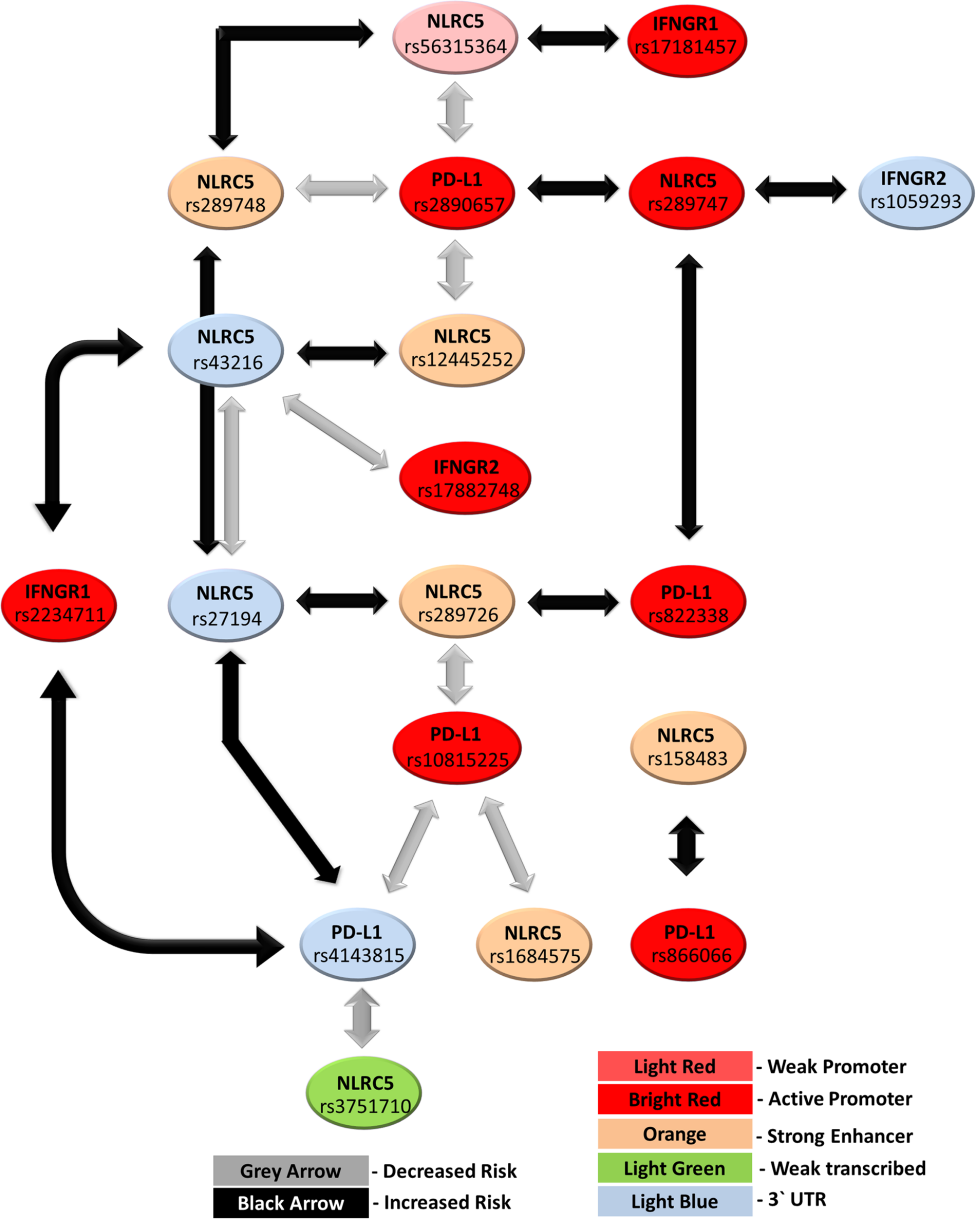
*NLRC5-PD-L1-IFNGR1/2* pair-wise interactions. The color indicates the SNPs’ location displayed by UCSC Genome Browser on lymphoblastoid cell lines (GM12878).

As shown in the Fig 1 most of the SNPs were interacting with two or more SNPs, either lying within the same or a different gene. Based on our selection criteria, the genotyped SNPs had pairwise LD r^2^ ≤ 0.80. However, some of the interactions can be explained by a lower level of LD (S1 Fig).

Three *NLRC5* SNPs, rs289747, rs289748 and rs56315364, mapping near/in the promoter (r^2^ = 0.42–0.70), showed an interaction with the same *PD-L1* promoter SNP rs2890657. Of note, we observed an increased risk of CRC development when at least one minor allele of rs2890657 interacted with the GG genotype of rs289747. Conversely, a protective effect was observed when the CC genotype of rs2890657 interacted with the CC genotype of rs289748 and rs56315364, respectively (Supplementary S5 Table).

On the other hand, the *PD-L1* SNP rs2890657 together with another promoter SNP, rs822338 (r^2^ = 0.68), interacted with the same *NLRC5* promoter SNP rs289747. Similar to rs2890657-rs289747 interaction, an increased risk of CRC was observed when at least one minor allele of rs822338 interacted with the GG genotype of rs289747 (S5 Table).

The two *PD-L1* promoter SNPs also interacted independently with two eQTL SNPs for *NLRC5*. The interaction partner for rs2890657 was rs12445252, whose T allele is predicted to decrease *NLRC5* expression in the whole blood tissue with an effect size of -0.26 and a p-value of 1.6e-7. rs822338 interacted with rs289726, whose C allele is reported to increase *NLRC5* expression in the whole blood with an effect size of 0.19 and a p-value of 10e-6. Interestingly, we observed a decreased CRC risk when the CC genotype of *PD-L1* rs2890657 interacted with at least one minor allele of *NLRC5* rs12445252, which is related with lower *NLRC5* expression and an increased CRC risk was shown by the interactions between the CC genotypes of both *PD-L1* rs822338 and *NLRC5* rs289726 and between the TT genotype of *PD-L1* rs822338 and the genotypes with at least one minor allele T of *NLRC5* rs289726 (S5 Table).

Two 3’UTR SNPs in *NLRC5*, rs43216 and rs27194, which map within a genetic block of 305 bp (r^2^ = 0.43), revealed a decreased risk of CRC when at least one major allele of rs27194 interacted with the minor allele genotype of rs43216. Additionally, both of them interacted independently with the two *NLRC5* eQTL SNPs, rs12445252 and rs289726, respectively. We observed an increased risk when the GG genotype of rs43216, which binds a lower number of miRNAs, interacted with the TT genotype of rs12445252, related to a lower expression of NLRC5, as well as when the TT genotype of rs27194, which also binds a lower number of miRNAs, interacted with the CC genotype of rs289726, which instead is related to a higher *NLRC5* expression. Moreover, the same genotype of rs27194 showed an increased risk also when it interacted with the CC genotype of the *NLRC5* flanking SNP rs289748 (S5 Table).

When we included the *IFNGR* genes variants previously analyzed in an older version of our cohort with a lower number of individuals (1327 cases and 758 healthy controls [17]) to our analysis, we observed 6 additional interactions including three SNPs within *IFNGR1* (rs2234711, rs1327474 and rs17181457) and two within *IFNGR2* (rs17882748 and rs1059293) (Table 3). Among them, rs2234711, lying within the 5’UTR of *IFNGR1*, showed complicated interactions with two 3’ UTR variants, *NLRC5* rs43216 and *PD-L1* rs4143815 (S6 Table). The strongest interaction was observed between a 3’UTR SNP in *IFNGR2* rs1059293 C>T and the *NLRC5* promoter SNP rs289747 G>A, which also survived the Bonferroni multiple testing correction (interaction term p value < 0.0004) and the FDR controlled at an arbitrary level q* < 0.10 (S4 Table). Particularly carriers of rs1059293 CC homozygous genotype and rs289747 heterozygous genotype showed an increased risk of CRC development (S6 Table). Curiously, an increased risk was also observed for T allele carriers of rs1059293 and GG genotype carriers of rs289747.

**Table 3:** *NLRC5-IFNGR1/2* and *PD-L1-IFNGR1/2* pair-wise interactions with cases and controls. Only the best genetic model of each SNP pair is shown.

SNP1	SNP2	Mode of inheritance SNP1	Mode of inheritance SNP2	LRT Statistic	DF	p-value based on LRT	LRT Statistic	DF	p-value based on LRT
				(interaction term)			(SNPs total)		
rs1059293	rs289747	Allele number	Three genotypes	21.17	2	**< .0001**	21.84	5	**0.001**
rs1059293	rs43216	Dominant	Three genotypes	9.64	2	**0.008**	11.64	5	**0.04**
rs17181457	rs56315364	Dominant	Recessive	3.95	1	**0.05**	12.53	3	**0.006**
rs17882748	rs43216	Recessive	Three genotypes	9.53	2	**0.009**	13.47	5	**0.019**
rs2234711	rs4143815	Dominant	Three genotypes	8.7	2	**0.013**	21.3	5	**0.001**
rs2234711	rs43216	Three genotypes	Dominant	7.72	2	**0.021**	19.19	5	**0.002**

DF: Degrees of Freedom

LTR: Likelihood Ratio Test

## Discussion

In our case-control study comprising up to 1424 cases and 1114 healthy controls, we investigated the role of genetic polymorphisms in the regulatory regions of *NLRC5*, *PD-L1* and the previously genotyped regulatory SNPs in the *IFNGR* genes on the risk of CRC. In the single SNP analysis, only 2 SNPs out of 16, rs1684575 T>G (OR: 1.60, 95% CI: 1.13–2.27, recessive model) and rs3751710 C>T (OR: 0.70, 95% CI: 0.51–0.96, dominant model), both mapping within the *NLRC5* gene, showed a nominal association with rectal cancer risk in the Czech population (p < 0.05). In our previous study on potentially functional *IFNGR* SNPs, rs2234711 in the 5’UTR of *IFNGR1*, was reported to be associated with an increased risk of CRC; particularly the risk allele C was associated with *IFNGR1* gene activity in a context-dependent manner [17,19].

Given that the evaluated 16 SNPs did not show a strong individual association with CRC risk and that SNPs represent common genetic alterations typically characterized by a low level of penetrance, we further evaluated whether their binary interactions might uncover synergistic effects contributing to CRC predisposition. Taking into account that all SNPs were non-coding variants, though located in regulatory regions (promoter, enhancer, 5´and 3´UTR), a possible biological mechanism may be an active involvement in the regulation of gene expression [20]. Perturbations in *NLRC5* and *PD-L1* gene expression may lead, as a consequence, to a dysregulation of the anti-tumor immune response, which in turn may influence CRC development [21–23]. Indeed, the immune infiltration is a major outcome factor in CRC [24,25] and altering immune-regulatory machinery is one of the mechanisms developed by cancer cells to evade the immune system and form a tumor [26,27].

Altogether, we observed 18 interactions between *NLRC5* and *PD-L1*, and further 6 interactions together with *IFNGR1/2* in a smaller sample set. For all interactions, the global null hypothesis test was highly significant (p-value < .0001). Twelve out of the 18 *PD-L1-NLRC5* interactions were below the threshold for the FDR controlled at an arbitrary level q*<0.10, while only one out of the 6 *PD-L1-NLRC5*-*IFNGR1/2* interactions survived both the FDR and the Bonferroni multiple testing correction (p-value< 0.0004). It should be pointed out that we had relatively low power to detect such an association in the first place, due to the limited number of cases in the interacting genotype categories and the high stringency of the Bonferroni correction [28]. Finally, the tests were not completely independent, due to the fact that many SNPs in each gene were studied and there was moderate to low LD between some of the SNPs.

The main interactions included three moderately linked *NLRC5* SNPs rs289747, rs289748, rs56315364, mapping within a genetic block of 3 kb located in the upstream and promoter region of the gene that exhibited a significant interaction with the same *PD-L1* promoter SNP rs2890657. Also another *PD-L1* promoter SNP rs822338 (r^2^ = 0.68 with rs2890657) interacted with the *NLRC5* promoter SNP rs289747. Interestingly, rs2890657 showed also an interaction with an eQTL SNP for *NLRC5*, rs12445252, while rs822338 interacted with another *NLRC5* eQTL SNP rs289726. Additionally, two 3’UTR SNPs in *NLRC5*, rs43216 and rs27194 (r^2^ = 0.43), interacted independently with the two *NLRC5* eQTL SNPs, and rs27194 also with another *NLRC5* flanking SNP rs289748. Furthermore, we observed interactions between a 5’ UTR SNP in *IFNGR1*, rs2234711, and 3’ UTR variants in *NLRC5* (rs43216) and *PD-L1* (rs4143815), respectively, and between a 3’ UTR SNP in *IFNGR2* (rs1059293) and a promoter SNP in *NLRC5* (rs289747).

All upstream and/or promoter SNPs in the *NLRC5* and *PD-L1* genes involved in the most significant interactions, and several other SNPs in high LD with them, are located within promoter histone marks and DNase hypersensivity sites. Two of the *NLRC5* SNPs, rs289747 and rs56315364, are predicted to affect in an opposite way the OCT proteins binding site, reflecting the opposite associations that they elicit on the CRC development and supporting the reliability of our interaction analysis. Also the *PD-L1* SNPs rs2890657 and rs822338 are estimated to affect transcription factor binding sites: rs2890657 the c-Myb binding site and rs822338 together with 5 linked SNPs the binding sites of transcription factors such as TAF1 (TATA-box binding protein associated factor 1) and p300. P300 is a histone acetyltransferase that regulates transcription of genes via chromatin remodeling [29]. Members of the TAF transcription factor family may participate in basal transcription, as coactivators, or in promoter recognition or to facilitate complex assembly and transcription initiation [30].

The two *PD-L1* promoter SNPs, rs2890657 and rs822338, also interacted independently with two *NLRC5* eQTL SNPs, rs12445252 and rs289726, respectively. Interestingly, we observed a lower CRC risk when *PD-L1* rs2890657 interacted with the allele of the *NLRC5* rs12445252, which is predicted to decrease *NLRC5* expression (-0.26 and a p-value of 1.6e-7), while the interaction between the different genotype categories of *PD-L1* rs822338 and *NLRC5* rs289726 was more complex, implicating an increased CRC risk for genotype combinations including rs289726 alleles predicted to either increase or decrease NLRC5 expression.

Furthermore, the two 3’UTR SNPs in *NLRC5*, rs43216 and rs27194 (r^2^ = 0.43), exhibited a decreased risk of CRC, which may be due to the interaction between the alleles that are predicted to bind a higher number of miRNAs than the other allele, leading to a stricter *NLRC5* post-transcriptional repression. Furthermore, both of them were found to be involved in an independent interaction with the two *NLRC5* eQTL SNPs. Particularly, an increased risk was observed when the rs43216 allele binding a lower number of miRNAs interacted with the rs12445252 allele related with a lower expression of *NLRC5*, as well as when the rs27194 allele which has a less strict post-transcriptional repression, interacted with the rs289726 allele related to a higher *NLRC5* expression, again reflecting the complex interactions between the genomic regions. Moreover, rs27194 showed an increased risk also when it interacted with *NLRC5* flanking SNP rs289748, when the allele of rs27194 binding lower number of miRNAs was involved in the interaction. These results suggest that a deregulation in the *NLRC5* expression through complicated interactions between genetic variants may lead to alterations in the downstream pathways and by that influence the risk of CRC.

Additionally, postulating that both, *NLRC5* and *PD-L1*, are downstream targets of IFNγ, we evaluated them in binary interaction with *IFNGR1* and *IFNGR2* variants previously genotyped by us [17]. We observed that the *IFNGR1* 5’UTR SNP, rs2234711, interacted with two 3’ UTR variants, *NLRC5* rs43216 and *PD-L1* rs4143815. The association between the *IFNGR1* SNP and the risk of CRC has already been established in our previous study [17]. In the present study the previous association was strengthened when the risk allele of rs2234711 interacted with the variant of rs43216, related with a stricter *NLRC5* post-transcriptional repression and with the allele of rs4143815 whose antisense is targeted by the miR-570, a negative regulator of *PD-L1*, as reported by the online prediction tools.

Finally, a 3’UTR SNP in *IFNGR2* rs1059293 C>T presented an interaction with the *NLRC5* promoter SNP rs289747 G>A. The result pointed to a complicated interaction between the two variants. An increased risk of CRC was observed both for carriers of the CC genotype for *IFNGR2* rs1059293 and the heterozygous GA genotype for *NLRC5* rs289747 and for the T allele carriers of rs1059293 and the GG genotype carriers of rs289747. In this context the C allele of rs1059293 has been reported to bind a lower number of miRNAs than the T allele. On the other hand, the *NLRC5* rs289747 is reported to affect OCT1 binding site, with the G allele showing a nearly inexistent affinity for OCT1, compared to the A allele, which instead is reported to exhibit a consistently increasing affinity. OCT1 is also reported to be overexpressed in many cancers, including CRC [31–33] and the IFNγ promoter has been reported to contain a binding site for Oct proteins [34]. As a consequence, the secretion of IFNγ by Oct proteins might be increased contributing to a dysregulation of the expression of the downstream pathway genes, such as *NLRC5* and *PD-L1* [35,36].

Assuming that NLRC5 has been reported to be the major MHC class I transactivator, a hyper-stimulation of its expression could lead to a strong CD8^+^ activation. Conversely a lower *NLRC5* expression has been reported to influence the MHC class I expression leading to an impaired ability to elicit CD8^+^ T-cell activation, which represent a way used by the tumor cells to escape the host immune system [23]. Additionally, recent data suggest that 5-Fluorouracil, a chemotherapeutic frequently used in CRC treatment, impacts on *PD-L1* expression [37]. Therefore the *PD-L1* SNPs studied here and their interactions with *IFNGR* and *NLRC5* variants may also be worth studying with regard to therapy response as well as survival of the CRC patients.

In this study, we included only four of the many immune-related genes for several reasons: first because of the interesting opposite effect that NLRC5 and PD-L1 exert on the regulation of T-cell mediated immunity, second because both of them are downstream targets of IFNγ and third because of the emerging role of these genes on CRC as well as on other cancer types. Furthermore, including a large network of genes would have led to a higher number of multiple tests, increasing the likelihood of chance findings. However, our study serves as a starting point to study the interplay between all the genes involved in the mucosal immune system, which would possibly shed light on the mechanisms underlying CRC development.

In conclusion, we anticipate that the interaction between the inherited genetic variants contributes to signaling defects, which in turn may lead to alteration in the anti-tumor immune response. Defects in the immune responses, especially in the expression of genes involved in immune surveillance, could favor tumorigenesis. Additionally, perturbation of the physiological immune homeostasis may also affect inflammation, another predisposing step for CRC development. It will be interesting to monitor the effect of the variants identified here under standard therapies for spontaneous and inflammation-related CRC and in ongoing clinical trials with immune check-point inhibitors where effects may be even more pronounced [16,37].

## Supporting information

S1 Table Information about the SNPs evaluated in this study provided by different online tools. (PDF)

S2 Table Association between the selected SNPs and the colorectal cancer risk. (PDF)

S3 Table False discovery rate for each individual *NLRC5-PD-L1* pair-wise interaction. (PDF)

S4 Table False discovery rate for each individual *NLRC5-PD-L1-IFNGR1/2* pair-wise interaction. (PDF)

S5 Table *NLRC5*-*PD-L1* pair-wise interactions. For the best model of each pair, age and sex adjusted ORs and 95% CI, with overall p-values based on the likelihood ratio test, were calculated.
(PDF)

S6 Table *IFNGR1/2*, *PD-L1* and *NLRC5* pair-wise interactions. For the best model of each pair, age and sex adjusted ORs and 95% CI, with overall p-values based on the likelihood ratio test, were calculated.
(PDF)

S1 Fig Haploview plot showing LD and haplotype blocks within *PD-L1* and *NLRC5* genes based on r^2^. (TIF)

## Abbreviations

AIC: Akaike information criterion
CI: confidence interval
CRC: Colorectal cancer
HWE: Hardy-Weinberg equilibrium
IBD: inflammatory bowel disease
IFN: interferon
IFNγ: Interferon gamma
IFNGR: interferon gamma receptor
LD: linkage disequilibrium
MHC: major histocompatibility complex
MAF: minor allele frequency
Nf-kB: nuclear factor kappa B
NLRC5: NLR family, CARD domain containing 5
NLRs: Nod-like receptors
OR: Odds ratio
PD-1: programmed death protein 1
PD-L1: programmed death-ligand 1
PRRs: pattern recognition receptors
SNPs: single nucleotide polymorphisms
TAF: TATA-box binding protein associated factor 1
TLRs: Toll-like receptors
TSS: transcription start site
UTR: untranslated regions
