# Supplementary material for: Investigation of single and synergic effects of NLRC5 and PD-L1 variants on the risk of colorectal cancer
Source: PLoS One. 2018 Feb 6;13(2):e0192385. doi: 10.1371/journal.pone.0192385 (PMC5800657; doi:10.1371/journal.pone.0192385)
Supplement: S1 Table — (PDF) [file pone.0192385.s001.pdf]

S1 Table. Information about the SNPs evaluated in this study provided by different online tools.

Haploreg								Ensembl	MicroSNiPer		GTex		Regulome
**Gene**	**SNP ID**	**Alleles**	**Position (hg38)**	**MAF (CEU)**	**linked SNPs**	**Proteins bound**	**Motifs changed**	**Regulatory features**	**MicroRNA binding site**		**Effect Size/ Tissue**	**p-value/ Gene**	**Chromatin State Digestive tract/ Blood & T-cell**
**NLRC5**	rs56315364	CT	16: 56987612	0.43	r^2>0.99 rs12598522<br>r^2>0.98 rs12149572<br>r^2>0.98 rs1566439	-	CACD, Irf, Klf4, MZF1::1-4, Pou2f2, RREB-1, Rad21, SP1, STAT, VDR, Zfp281	Promoter flanking region	-	-	-	-	Weak Repressed PolyComb/Weak transcription<br>Flanking Active TSS/Enhancers
**NLRC5**	rs289747	GA	16: 56990026	0.36	No LD	ZNF263	Bbx, Gmeb1, Pou2f2, Oct1	Promoter	-	-	-	-	Active TSS/Flanking Active TSS<br>Active TSS/Flanking Active TSS
**NLRC5**	rs7197864	GA	16: 56990972	0.11	r^2=1 rs7205459<br>r^2>0.99 rs17290922<br>r^2>0.99 rs12924030	PAX5C20, POL2, POL24H8, TBP, MAX	E2F, Smad3	Promoter flanking region	-	-	-	-	Weak transcription/Flanking Active TSS/Enhancers<br>Active TSS/Flanking Active TSS
**NLRC5**	rs289748	CT	16: 56991151	0.48	No LD	TBP	Maf, NRSF	Promoter flanking region	-	-	-	-	Flanking Active TSS/Weak transcription/Enhancers<br>Active TSS/Flanking Active TSS
**NLRC5**	rs1684575	GT	16: 57023707	0.56	r^2 >0.95 rs1672865	-	BCL, Ets, NF-kB, STAT	intronic	-	-	0.25 Whole Blood	7.50E-10 NLRC5	Strong/Weak transcription<br>Genic Enhancers/Flanking Active TSS/Enhancers
**NLRC5**	rs12445252	CT	16: 57023767	0.28	r^2= 1 rs12444396<br>r^2> 0.97 rs4784750<br>r^2> 0.97 rs4784751	-	CCNT2, EWSR1-FLI1, GATA, Pax-4, TATA	intronic eQTL	-	-	-0.26 Whole Blood	1.60E-07 NLRC5	Strong/Weak transcription<br>Genic enhancers/Flanking Active TSS/Enhancers
**NLRC5**	rs289726	TC	16: 57040539	0.65	r^2 >0.94 rs289727	-	Brachyury, ERα-a, Esr2, HNF6	intronic eQTL	-	-	0.19 Whole Blood	0.00001 NLRC5	Strong transcription/Genic enhancers<br>Genic enhancers/Flanking Active TSS/Enhancers
**NLRC5**	rs158483	CT	16: 57042785	0.27	r^2 >0.89 rs289723	POL2	BRCA1, Osr	Intron variant	-	-	-	-	Strong transcription/Genic enhancers<br>Genic enhancers/Flanking Active TSS/Enhancers
**NLRC5**	rs3751710	CT	16: 57061863	0.16	r^2 >0.98 rs58239985	-	STAT, Smad	Promoter flanking region	-	-	-	-	Strong/Weak transcription/Genic enhancers<br>Strong transcription
**NLRC5**	rs27194	AT	16: 57082602	0.76	r^2=1 rs27195	-	AP-1, AP-2, BAF155, BATF, BCL, Bach1, Bsx, Dlx2, Dlx5, GR, Irf, KAP1, Maf, Myc, Nrf-2, PRDM1, Prrx2, STAT, TCF4, p300	3´ UTR variant	miR-4729<br>miR-616-3p<br>miR-5696	miR-942<br>miR-4251	-	-	Strong /Weak transcription<br>Strong transcription
**NLRC5**	rs43216	GA	16: 57082907	0.64	r^2>0.94 rs150348<br>r^2>0.94 rs154042<br>r^2>0.92 rs154045	-	BCL, Ets, NF-kappaB, STAT	3´ UTR variant	miR-96-3p<br>miR-3614-5p<br>+ other 5 miRNAs	miR-96-3p<br>miR-4793-5p<br>+ other 7 miRNAs	-	-	Strong /Weak transcription<br>Strong transcription

S1 Table. cont.

Gene	SNP ID	Alleles	Position (hg38)	MAF (CEU)	linked SNPs	Proteins bound	Motifs changed	Regulatory features	MicroRNA binding site		Effect Size/ Tissue	p-value/ Gene	Chromatin State Digestive tract/ Blood & T-cell
**5bp 5' of CD274**	rs10815225	GC	9: 5450497	0.12	r^2 >0.89 rs10975121	POL2, CTCF, NFKB, EBF1, ELF1, NRF1, OCT2, PAX5C20, POL24H8, POU2F2, RAD21, SP1, TAF1, TBP, TCF12, YY1, ZEB1, NANOG, TCF4, POL2B, AP2α, AP2γ, CEBPB, SMC3, HDAC2, E2F6, EGR1, HMGN3, MAX, ZBTB7A, HAE2F1, CMYC	AP-1, AP-2, CHD2, ELF1, Egr-1, Ets, Hic1, Irf, Klf7, SP1, Sp4, Znf143	Promoter	-	-	0.43<br>Esophagus - Mucosa	0.000032<br>CD274	Active TSS<br>Active TSS/Flanking Active TSS
**CD274**	rs866066	CT	9: 5450953	0.42	r^2 >0.96 rs12002985<br>r^2 >0.95 rs4742097	POL2	AP-4, E2A, HDAC2, Lmo2-complex, MIF-1, Maf, Myf, NRSF, Pou2f2, RP58, RXRA, Rad21, SMC3, Sin3Ak-20, TAL1	Promoter	-	-	-0.20<br>Whole Blood	1.90E-08<br>CD274	Active TSS/Flanking Active TSS/Weak transcription<br>Active TSS/Flanking Active TSS
**CD274**	rs822338	TC	9: 5451557	0.7	r^2 >0.96 rs860290<br>r^2 >0.96 rs822340<br>r^2 >0.96 rs822341<br>r^2 >0.96 rs822342<br>r^2 >0.96 rs17742278<br>plus others 3 SNPs	POL2, TBP, USF2	Sox	Promoter	-	-	-	-	Quiescent/Low/Enhancer/Weak transcription<br>Active TSS/Flanking Active TSS/Enhancers
**CD274**	rs2890657	GC	9: 5452560	0.23	r^2 >0.96 rs10815226<br>r^2 >0.95 rs10481593<br>r^2 >0.95 rs6651524<br>r^2 >0.95 rs6651525	EBF1	Myb	Promoter flanking region	-	-	-	-	Quiescent/Low/Weak transcription<br>Transcr. at gene 5' and 3'/Enhancers
**CD274**	rs4143815	GC	9: 5468257	0.33	No LD	-	Foxo, Irf, Pax-5, Pax-6, RXRA, Sox, p300	3´ UTR variant	miR-570-3p	miR-539-5p	-	-	Quiescent/Low/Strong /Weak transcription<br>Strong transcription

S1 Table. cont.

Gene	SNP ID	Alleles	Position (hg38)	MAF (CEU)	linked SNPs	Proteins bound	Motifs changed	Regulatory features	MicroRNA binding site		Effect Size/ Tissue	p-value/ Gene	Chromatin State<br>Digestive tract/ Blood & T-cell
**IFNRG1**	rs2234711	TC	6: 137540520	0.39	$r^2 = 1$ rs7749390	POL2	-	5´ UTR variant	-	-	-	-	Active TSS<br>Active TSS/Flanking Active TSS/Transcr. at gene 5' and 3'
**IFNRG1**	rs17181457	CT	6: 137540536	0.08	No LD	POL2,EGR1,GABP, OCT2,POU2F2,SP1, TBP,POL24H8,TAF1 ,PAX5C20,KAP1,ELF 1,HEY1,JUND,TR4,C CNT2,CMYC,E2F6,H MGN3,IRF1,MAX,Y Y1,STAT3	BCL,CAC-binding-protein,E2F,EBF,N RSF,Sin3Ak-20,Zfx,Znf143	5´ UTR variant	-	-	-	-	Active TSS<br>Active TSS/Flanking Active TSS/Transcr. at gene 5' and 3'
**IFNRG1**	rs1327474	GA	6: 137541075	0.55	No LD	POL2	-	Upstream 5´ gene variant	-	-	-	-	Weak transcription/Flanking Active TSS/Quiescent<br>Flanking Active TSS/Enhancers
**IFNRG2**	rs17882748	TC	21: 34775721	0.48	No LD	POL2,EGR1,YY1,NF KB,TAF1,POL24H8, HAE2F1,HEY1,CCN T2	BCL,BHLHE40,HEY 1,Myc,Pou2f2,Sin3 Ak-20,YY1	5´ UTR variant	-	-	-	-	Active TSS<br>Active TSS/Bivalent Enhancers
**IFNRG2**	rs1059293	TC	21: 34809693	0.46	$r^2 > 0.90$ with more than 25 SNPs	-	-	3´ UTR variant	miR-4646-3p<br>miR-512-5p<br>+ other 15 miRNAs	miR-4252<br>miR-3177-5p<br>+ other 3 miRNAs	-0.47	2.70E-28<br>TMEM50B	Strong /Weak transcription<br>Strong /Weak transcription/Genetic Enhancer
