# Supplementary material for: Investigation of single and synergic effects of NLRC5 and PD-L1 variants on the risk of colorectal cancer
Source: PLoS One. 2018 Feb 6;13(2):e0192385. doi: 10.1371/journal.pone.0192385 (PMC5800657; doi:10.1371/journal.pone.0192385)
Supplement: S2 Table — (PDF) [file pone.0192385.s002.pdf]

S2 Table. Association between the selected SNPs and the colorectal cancer risk

Gene	SNP ID	Overall					Colon				Rectum			
		Genotype	Cases	Controls	OR (95% CI)	*P* value	Cases	Controls	OR (95% CI)	P Val	Cases	Controls	OR (95% CI)	*P* value
NLRC5	rs27194_AT	A/A	841(61.61%)	652(59.43%)	1.00		508(61.28%)	652(59.43%)	1.00		225(61.48%)	652(59.43%)	1.00	
		A/T	436(31.94%)	387(35.28%)	0.89(0.72-1.10)	0.27	274(33.05%)	387(35.28%)	0.92(0.72-1.16)	0.46	113(30.87%)	387(35.28%)	0.83(0.61-1.13)	0.24
		T/T	88(6.45%)	58(5.29%)	1.24(0.80-1.90)	0.34	47(5.67%)	58(5.29%)	1.02(0.62-1.69)	0.94	28(7.65%)	58(5.29%)	1.24(0.70-2.21)	0.46
	rs1684575_GT	T/T	467(35.43%)	410(37.31%)	1.00		289(35.95%)	410(37.31%)	1.00		129(36.24%)	410(37.31%)	1.00	
		G/T	596(45.22%)	512(46.59%)	0.98(0.79-1.22)	0.87	369(45.90%)	512(46.59%)	1.03(0.81-1.32)	0.81	148(41.57%)	512(46.59%)	0.97(0.71-1.33)	0.85
		**G/G**	255(19.35%)	177(16.11%)	1.19(0.89-1.58)	0.24	146(18.16%)	177(16.11%)	1.12(0.81-1.55)	0.49	**79(22.19%)**	**177(16.11%)**	**1.57(1.06-2.32)**	**0.024**
	**Recessive Model**	T/T+ G/T	1063(80.65%)	922(83.89%)	1.00		658(81.84%)	922(83.89%)	1.00		277(77.81%)	922(83.89%)	1.00	
		**G/G**	255(19.35%)	177(16.11%)	1.20(0.93-1.55)	0.17	146(18.16%)	177(16.11%)	1.10(0.82-1.48)	0.52	**79(22.19%)**	**177(16.11%)**	**1.60(1.13-2.27)**	**0.009**
	rs43216_AG	G/G	540(40.54%)	417(38.05%)	1.00		325(40.32%)	417(38.05%)	1.00		149(41.16%)	417(38.05%)	1.00	
		A/G	593(44.52%)	508(46.35%)	0.93(0.75-1.15)	0.50	366(45.41%)	508(46.35%)	0.95(0.74-1.20)	0.65	158(43.65%)	508(46.35%)	0.91(0.67-1.23)	0.53
		A/A	199(14.94%)	171(15.60%)	0.85(0.63-1.14)	0.27	115(14.27%)	171(15.60%)	0.82(0.58-1.15)	0.25	55(15.19%)	171(15.60%)	0.84(0.55-1.28)	0.42
	rs3751710_CT	C/C	966(70.20%)	765(69.42%)	1.00		574(68.82%)	765(69.42%)	1.00		277(74.26%)	765(69.42%)	1.00	
		**C/T**	358(26.02%)	303(27.50%)	0.88(0.71-1.10)	0.26	229(27.46%)	303(27.50%)	0.94(0.73-1.20)	0.63	**83(22.25%)**	**303(27.50%)**	**0.66(0.48-0.92)**	**0.014**
		T/T	52(3.78%)	34(3.09%)	1.30(0.76-2.21)	0.34	31(3.72%)	34(3.09%)	1.36(0.75-2.48)	0.31	13(3.49%)	34(3.09%)	1.02(0.48-2.19)	0.95
	**Dominant Model**	**C/T+T/T**	410(29.80%)	337(30.58%)	0.92(0.75-1.14)	0.44	260(31.18%)	337(30.58%)	0.98(0.77-1.24)	0.87	**96(25.74%)**	**337(30.58%)**	**0.70(0.51-0.96)**	**0.025**
	rs7197864_GA	G/G	1043(79.14%)	856(78.89%)	1.00		634(79.45%)	856(78.89%)	1.00		274(77.18%)	856(78.89%)	1.00	
		G/A	248(18.82%)	212(19.54%)	0.90(0.70-1.15)	0.40	146(18.30%)	212(19.54%)	0.85(0.64-1.13)	0.27	73(20.56%)	212(19.54%)	0.95(0.67-1.34)	0.77
		A/A	27(2.05%)	17(1.57%)	1.10(0.53-2.29)	0.80	18(2.26%)	17(1.57%)	1.25(0.56-2.78)	0.59	8(2.25%)	17(1.57%)	1.35(0.49-3.70)	0.56
	rs289748_TC	C/C	369(27.31%)	300(27.35%)	1.00		224(27.15%)	300(27.35%)	1.00		96(26.45%)	300(27.35%)	1.00	
		T/C	652(48.26%)	542(49.41%)	0.95(0.76-1.20)	0.69	402(48.73%)	542(49.41%)	0.97(0.75-1.26)	0.83	181(49.86%)	542(49.41%)	0.96(0.69-1.35)	0.84
		T/T	330(24.43%)	255(23.25%)	1.01(0.77-1.33)	0.94	199(24.12%)	255(23.25%)	1.03(0.76-1.40)	0.84	86(23.69%)	255(23.25%)	1.03(0.70-1.53)	0.87
	rs12445252_CT	C/C	718(53.90%)	586(53.76%)	1.00		446(55.13%)	586(53.76%)	1.00		195(53.42%)	586(53.76%)	1.00	
		C/T	500(37.54%)	428(39.27%)	0.96(0.78-1.17)	0.67	298(36.84%)	428(39.27%)	0.94(0.75-1.19)	0.63	137(37.53%)	428(39.27%)	1.04(0.77-1.40)	0.80
		T/T	114(8.56%)	76(6.97%)	1.06(0.72-1.54)	0.77	65(8.03%)	76(6.97%)	1.00(0.65-1.54)	1.00	33(9.04%)	76(6.97%)	1.37(0.82-2.28)	0.24

S2 Table. cont.

		Genotype	Cases	Controls	OR (95% CI)	*P* value	Cases	Controls	OR (95% CI)	*P* value	Cases	Controls	OR (95% CI)	*P* value
NLRC5	rs289747_GA	G/G	530(39.20%)	397(36.36%)	1.00		323(39.06%)	397(36.36%)	1.00		146(40.67%)	397(36.36%)	1.00	
		G/A	616(45.56%)	532(48.72%)	0.93(0.75-1.14)	0.47	380(45.95%)	532(48.72%)	0.95(0.75-1.21)	0.67	159(44.29%)	532(48.72%)	0.83(0.61-1.13)	0.23
		A/A	206(15.24%)	163(14.93%)	0.96(0.71-1.29)	0.78	124(14.99%)	163(14.93%)	0.96(0.69-1.34)	0.80	54(15.04%)	163(14.93%)	0.99(0.65-1.52)	0.98
	rs56315364_CT	C/C	474(35.75%)	389(35.56%)	1.00		282(34.94%)	389(35.56%)	1.00		132(36.57%)	389(35.56%)	1.00	
		T/C	620(46.76%)	541(49.45%)	0.92(0.74-1.14)	0.42	382(47.34%)	541(49.45%)	0.94(0.74-1.21)	0.64	169(46.81%)	541(49.45%)	0.86(0.63-1.17)	0.35
		T/T	232(17.50%)	164(14.99%)	1.24(0.92-1.65)	0.15	143(17.72%)	164(14.99%)	1.25(0.90-1.74)	0.17	60(16.62%)	164(14.99%)	1.22(0.81-1.84)	0.34
	rs289726_CT	C/C	571(41.74%)	460(41.63%)	1.00		348(42.03%)	460(41.63%)	1.00		154(41.40%)	460(41.63%)	1.00	
		C/T	612(44.74%)	502(45.43%)	0.98(0.80-1.21)	0.87	374(45.17%)	502(45.43%)	1.06(0.84-1.34)	0.63	160(43.01%)	502(45.43%)	0.99(0.73-1.34)	0.95
		T/T	185(13.52%)	143(12.94%)	1.06(0.78-1.44)	0.71	106(12.80%)	143(12.94%)	1.05(0.74-1.50)	0.77	58(15.59%)	143(12.94%)	1.34(0.88-2.04)	0.18
	rs158483_CT	C/C	700(52.20%)	548(49.82%)	1.00		421(51.40%)	548(49.82%)	1.00		185(51.68%)	548(49.82%)	1.00	
		C/T	532(39.67%)	463(42.09%)	0.91(0.74-1.11)	0.35	325(39.68%)	463(42.09%)	0.90(0.71-1.13)	0.37	148(41.34%)	463(42.09%)	0.80(0.60-1.08)	0.15
		T/T	109(8.13%)	89(8.09%)	1.22(0.85-1.76)	0.28	73(8.91%)	89(8.09%)	1.30(0.87-1.95)	0.20	25(6.98%)	89(8.09%)	1.20(0.70-2.05)	0.50
PD-L1	rs10815225_CG	G/G	1003(74.02%)	850(77.34%)	1.00		620(75.06%)	850(77.34%)	1.00		261(71.31%)	850(77.34%)	1.00	
		C/G	340(25.09%)	237(21.57%)	1.17(0.93-1.47)	0.17	201(24.33%)	237(21.57%)	1.09(0.84-1.42)	0.50	99(27.05%)	237(21.57%)	1.30(0.95-1.79)	0.10
		C/C	12(0.89%)	12(1.09%)	0.39(0.13-1.14)	0.09	**5(0.61%)**	**12(1.09%)**	**0.18(0.05-0.66)**	**0.01**	6(1.64%)	12(1.09%)	0.74(0.21-2.60)	0.63
	rs822338_CT	T/T	703(52.07%)	561(51.00%)	1.00		410(50.00%)	561(51.00%)	1.00		202(55.04%)	561(51.00%)	1.00	
		C/T	528(39.11%)	435(39.55%)	1.03(0.84-1.27)	0.78	345(42.07%)	435(39.55%)	1.17(0.93-1.48)	0.18	124(33.79%)	435(39.55%)	0.92(0.68-1.25)	0.60
		C/C	119(8.81%)	104(9.45%)	0.95(0.67-1.35)	0.79	65(7.93%)	104(9.45%)	0.95(0.64-1.43)	0.82	41(11.17%)	104(9.45%)	1.20(0.75-1.91)	0.45
	rs866066_CT	C/C	414(30.80%)	302(27.61%)	1.00		231(28.27%)	302(27.61%)	1.00		127(34.89%)	302(27.61%)	1.00	
		C/T	632(47.02%)	537(49.09%)	0.93(0.74-1.17)	0.55	400(48.96%)	537(49.09%)	1.04(0.80-1.35)	0.77	164(45.05%)	537(49.09%)	0.86(0.62-1.18)	0.35
		T/T	298(22.17%)	255(23.31%)	0.90(0.69-1.19)	0.46	186(22.77%)	255(23.31%)	0.94(0.69-1.28)	0.69	73(20.05%)	255(23.31%)	0.74(0.50-1.10)	0.14
	rs2890657_CG	G/G	825(61.07%)	667(60.64%)	1.00		492(59.78%)	667(60.64%)	1.00		231(63.29%)	667(60.64%)	1.00	
		C/G	452(33.46%)	368(33.45%)	1.05(0.85-1.29)	0.67	290(35.24%)	368(33.45%)	1.14(0.90-1.44)	0.29	106(29.04%)	368(33.45%)	0.89(0.65-1.21)	0.44
		C/C	74(5.48%)	65(5.91%)	0.89(0.58-1.35)	0.58	41(4.98%)	65(5.91%)	0.86(0.53-1.41)	0.55	28(7.67%)	65(5.91%)	1.24(0.72-2.16)	0.44
	rs4143815_CG	G/G	632(46.47%)	514(46.60%)	1.00		388(47.09%)	514(46.60%)	1.00		167(45.01%)	514(46.60%)	1.00	
		C/G	580(42.65%)	467(42.34%)	1.03(0.84-1.26)	0.80	345(41.87%)	467(42.34%)	1.07(0.85-1.35)	0.58	162(43.67%)	467(42.34%)	1.00(0.75-1.34)	0.98
		C/C	148(10.88%)	122(11.06%)	0.87(0.63-1.20)	0.40	91(11.04%)	122(11.06%)	0.89(0.62-1.29)	0.55	42(11.32%)	122(11.06%)	0.87(0.55-1.39)	0.57
