# Supplementary material for: Investigation of single and synergic effects of NLRC5 and PD-L1 variants on the risk of colorectal cancer
Source: PLoS One. 2018 Feb 6;13(2):e0192385. doi: 10.1371/journal.pone.0192385 (PMC5800657; doi:10.1371/journal.pone.0192385)
Supplement: S3 Table — (PDF) [file pone.0192385.s003.pdf]

S3 Table. False discovery rate for each individual *NLRC5-PD-L1* pair-wise interaction

SNP1	SNP2	Mode of inheritance SNP1	Mode of inheritance SNP2	p-value based on LR test interaction term	p-value based on LR test SNPs total	q*
**rs27194_AT**	**rs289726_CT**	**Three genotypes**	**Dominant**	**0.0015**	**0.008**	**0.09**
**rs12445252_CT**	**rs43216_AG**	**Recessive**	**Dominant**	**0.0022**	**0.01**	**0.09**
**rs289726_CT**	**rs822338_CT**	**Dominant**	**Three genotypes**	**0.0028**	**0.03**	**0.09**
**rs2890657_CG**	**rs289747_GA**	**Dominant**	**Dominant**	**0.0048**	**0.036**	**0.09**
**rs2890657_CG**	**rs56315364_CT**	**Recessive**	**Dominant**	**0.0053**	**0.045**	**0.09**
**rs10815225_CG**	**rs289726_CT**	**Recessive**	**Dominant**	**0.0054**	**0.011**	**0.09**
**rs12445252_CT**	**rs2890657_CG**	**Dominant**	**Recessive**	**0.0056**	**0.048**	**0.09**
**rs27194_AT**	**rs289748_TC**	**Recessive**	**Dominant**	**0.0058**	**0.04**	**0.09**
**rs2890657_CG**	**rs289748_TC**	**Recessive**	**Dominant**	**0.0072**	**0.04**	**0.09**
**rs289747_GA**	**rs822338_CT**	**Dominant**	**Dominant**	**0.0083**	**0.046**	**0.09**
**rs10815225_CG**	**rs4143815_CG**	**Recessive**	**Recessive**	**0.0088**	**0.009**	**0.09**
rs1684575_GT	rs2890657_CG	Dominant	Recessive	0.01	0.07	0.09
**rs27194_AT**	**rs4143815_CG**	**Recessive**	**Dominant**	**0.01**	**0.05**	**0.09**
**rs3751710_CT**	**rs4143815_CG**	**Three genotypes**	**Recessive**	**0.013**	**0.04**	**0.11**
rs289748_TC	rs822338_CT	Recessive	Recessive	0.017	0.10	0.12
rs158483_CT	rs289726_CT	Dominant	Recessive	0.0172	0.12	0.12
**rs158483_CT**	**rs866066_CT**	**Recessive**	**Recessive**	**0.0173**	**0.05**	**0.12**
rs158483_CT	rs289748_TC	Three genotypes	Dominant	0.0174	0.07	0.12
rs289747_GA	rs3751710_CT	Three genotypes	Recessive	0.02	0.10	0.12
rs158483_CT	rs7197864_GA	Recessive	Dominant	0.021	0.06	0.12
rs12445252_CT	rs7197864_GA	Dominant	Dominant	0.022	0.14	0.12
rs1684575_GT	rs7197864_GA	Recessive	Dominant	0.026	0.09	0.14
rs289726_CT	rs289747_GA	Dominant	Recessive	0.028	0.18	0.15
rs158483_CT	rs1684575_GT	Recessive	Dominant	0.03	0.09	0.16
**rs27194_AT**	**rs56315364_CT**	**Recessive**	**Three genotypes**	**0.034**	**0.04**	**0.16**
rs2890657_CG	rs289726_CT	Dominant	Dominant	0.036	0.22	0.16
**rs27194_AT**	**rs43216_AG**	**Recessive**	**Recessive**	**0.0361**	**0.03**	**0.16**
rs1684575_GT	rs3751710_CT	Dominant	Recessive	0.04	0.16	0.17
rs3751710_CT	rs866066_CT	Dominant	Recessive	0.041	0.19	0.17
**rs289748_TC**	**rs56315364_CT**	**Recessive**	**Recessive**	**0.042**	**0.02**	**0.17**
rs289726_CT	rs866066_CT	Recessive	Three genotypes	0.046	0.23	0.17
rs2890657_CG	rs4143815_CG	Recessive	Dominant	0.047	0.22	0.17
**rs10815225_CG**	**rs1684575_GT**	**Recessive**	**Three genotypes**	**0.048**	**0.03**	**0.17**
rs866066_CT	rs289748_TC	Recessive	Recessive	0.052	0.20	0.18
rs27194_AT	rs289747_GA	Dominant	Dominant	0.056	0.21	0.18
rs3751710_CT	rs10815225_CG	Dominant	Dominant	0.057	0.15	0.18
rs289748_TC	rs1684575_GT	Dominant	Recessive	0.057	0.13	0.18

S3 Table. cont.

rs1684575_GT	rs56315364_CT	Recessive	Recessive	0.06	0.02	0.19
rs289726_CT	rs12445252_CT	Dominant	Recessive	0.066	0.32	0.20
rs27194_AT	rs1684575_GT	Recessive	Recessive	0.069	0.09	0.20
rs822338_CT	rs27194_AT	Recessive	Recessive	0.0693	0.22	0.20
rs866066_CT	rs822338_CT	Dominant	Recessive	0.074	0.23	0.21
rs3751710_CT	rs12445252_CT	Recessive	Dominant	0.076	0.26	0.21
rs43216_AG	rs289747_GA	Dominant	Dominant	0.0765	0.21	0.21
rs289748_TC	rs289747_GA	Recessive	Dominant	0.077	0.31	0.21
rs27194_AT	rs2890657_CG	Recessive	Recessive	0.079	0.16	0.21
rs866066_CT	rs4143815_CG	Dominant	Dominant	0.087	0.35	0.22
rs822338_CT	rs12445252_CT	Recessive	Dominant	0.090	0.40	0.22
rs289747_GA	rs4143815_CG	Dominant	Recessive	0.091	0.25	0.22
rs27194_AT	rs10815225_CG	Dominant	Recessive	0.092	0.07	0.22
rs43216_AG	rs3751710_CT	Recessive	Dominant	0.097	0.24	0.23
rs289748_TC	rs3751710_CT	Recessive	Recessive	0.10	0.34	0.24
rs866066_CT	rs10815225_CG	Recessive	Dominant	0.108	0.29	0.24
rs289747_GA	rs10815225_CG	Dominant	Recessive	0.115	0.09	0.25
rs43216_AG	rs1684575_GT	Dominant	Recessive	0.116	0.19	0.25
rs1684575_GT	rs289726_CT	Recessive	Recessive	0.12	0.27	0.25
rs10815225_CG	rs12445252_CT	Recessive	Dominant	0.122	0.11	0.25
rs158483_CT	rs12445252_CT	Recessive	Dominant	0.124	0.26	0.25
rs1684575_GT	rs4143815_CG	Recessive	Recessive	0.126	0.28	0.25
rs822338_CT	rs2890657_CG	Dominant	Recessive	0.127	0.31	0.25

Interaction with a likelihood ratio test-based P-values < 0.05 both for interaction term and SNP total are highlighted in bold font

$q^{*} = mP_{(1)}/i$
