# Supplementary material for: Investigation of single and synergic effects of NLRC5 and PD-L1 variants on the risk of colorectal cancer
Source: PLoS One. 2018 Feb 6;13(2):e0192385. doi: 10.1371/journal.pone.0192385 (PMC5800657; doi:10.1371/journal.pone.0192385)
Supplement: S4 Table — (PDF) [file pone.0192385.s004.pdf]

S4 Table. False discovery rate for each individual *NLRC5-PD-L1-IFNGR1/2* pair-wise interaction

SNP1	SNP2	Mode of inheritance SNP1	Mode of inheritance SNP2	p-value based on LR test interaction term	p-value based on LR test SNPs total	q*
**rs1059293_CT**	**rs289747_GA**	**Allele number**	**Three genotypes**	**0.0001**	**0.0006**	**0.01**
**rs1059293_CT**	**rs43216_AG**	**Dominant**	**Three genotypes**	**0.0081**	**0.04**	**0.14**
**rs17882748_CT**	**rs43216_AG**	**Recessive**	**Three genotypes**	**0.0085**	**0.02**	**0.14**
rs1059293_CT	rs289748_TC	Allele number	Recessive	0.009	0.08	0.14
rs17882748_CT	rs4143815_CG	Allele number	Allele number	0.0098	0.06	0.14
rs17882748_CT	rs822338_CT	Allele number	Allele number	0.011	0.06	0.14
**rs2234711_CT**	**rs4143815_CG**	**Dominant**	**Three genotypes**	**0.013**	**0.001**	**0.15**
rs17882748_CT	rs2890657_CG	Allele number	Dominant	0.017	0.08	0.16
rs1327474_AG	rs4143815_CG	Allele number	Dominant	0.018	0.09	0.16
**rs2234711_CT**	**rs43216_AG**	**Three genotypes**	**Dominant**	**0.021**	**0.002**	**0.17**
rs1059293_CT	rs2890657_CG	Dominant	Allele number	0.038	0.22	0.25
rs17181457_CT	rs27194_AT	Three genotypes	Dominant	0.046	0.06	0.25
rs17181457_CT	rs289748_TC	Recessive	Recessive	0.046	0.26	0.25
**rs17181457_CT**	**rs56315364_CT**	**Dominant**	**Recessive**	**0.047**	**0.006**	**0.25**
rs17882748_CT	rs866066_CT	Allele number	Dominant	0.0472	0.13	0.25

Interaction with a likelihood ratio test-based P-values < 0.05 both for interaction term and SNP total are highlighted in bold font

q*= $mP_{(1)}/i$
