# Supplementary material for: Investigation of single and synergic effects of NLRC5 and PD-L1 variants on the risk of colorectal cancer
Source: PLoS One. 2018 Feb 6;13(2):e0192385. doi: 10.1371/journal.pone.0192385 (PMC5800657; doi:10.1371/journal.pone.0192385)
Supplement: S5 Table — For the best model of each pair, age and sex adjusted ORs and 95% CI, with overall p-values based on the likelihood ratio test, were calculated. (PDF) [file pone.0192385.s005.pdf]

S5 Table. ***NLRC5-PD-L1* pair-wise interactions.** For the best model of each pair, age and sex adjusted ORs and 95% CI, with overall p-values based on the likelihood ratio test, were calculated.

PD-L1 rs2890657	95% CI P-Value	*NLRC5* rs289747 GG	*NLRC5* rs289747 GA+AA
	GG	1.00	1.17 (0.91-1.52) (0.22)
	GC+CC	1.52 (1.09-2.12) **(0.014)**	0.98 (0.74-1.29) (0.86)

Overall p-value: **0.036**

PD-L1 rs2890657	95% CI P-Value	*NLRC5* rs289748 CC	*NLRC5* rs289748 CT+TT
	GG+GC	1.00	0.88 (0.70-1.11) (0.29)
	CC	0.33 (0.15-0.74) **(0.007)**	1.07 (0.62-1.84) (0.82)

Overall p-value: **0.04**

PD-L1 rs2890657	95% CI P-Value	*NLRC5* rs56315364 CC	*NLRC5* rs56315364 CT+TT
	GG+GC	1.00	0.91 (0.74-1.13) (0.39)
	CC	0.41 (0.20-0.84) **(0.014)**	1.33 (0.76-2.32) (0.33)

Overall p-value: **0.045**

PD-L1 rs822338	95% CI P-Value	*NLRC5* rs289747 GG	*NLRC5* rs289747 GA+AA
	TT	1.00	1.18 (0.89-1.57) (0.25)
	CT+CC	1.46 (1.06-2.01) **(0.021)**	1.00 (0.75-1.32) (0.98)

Overall p-value: **0.046**

NLRC5 rs12445252	95% CI P-Value	*PD-L1* rs2890657 GG+CG	*PD-L1* rs2890657 CC
	CC	1.00	1.63 (0.88-3.01) (0.12)
	CT +TT	1.08 (0.88-1.32) (0.48)	0.52 (0.28-0.96) **(0.036)**

Overall p-value: **0.048**

NLRC5 rs289726	95% CI P-Value	*PD-L1* rs822338 TT	*PD-L1* rs822338 CT	*PD-L1* rs822338 CC
	CC	1.00	1.32 (0.96-1.83) (0.09)	1.86 (1.07-3.23) **(0.029)**
	CT+TT	1.36 (1.03-1.80) **(0.032)**	1.17 (0.87-1.58) (0.30)	0.78 (0.49-1.25) (0.31)

Overall p-value: **0.033**

S5 Table. cont.

NLRC5 rs27194	95% CI P-Value	*NLRC5* rs43216	
		GG+AG	AA
	AA+AT	1.00	0.68 (0.49-0.96) **(0.026)**
	TT	0.42 (0.11-1.61) (0.021)	1.36 (0.86-2.15) (0.19)

Overall p-value: **0.033**

NLRC5 rs27194	95% CI P-Value	*NLRC5* rs289726	
		CC	CT+TT
	AA	1.00	0.94 (0.73 -1.21) (0.64)
	AT	0.69 (0.49-0.97) **(0.031)**	0.97 (0.74-1.30) (0.89)
	TT	3.11 (1.40-6.95) **(0.006)**	0.77 (0.45-1.32) (0.34)

Overall p-value: **0.008**

NLRC5 rs12445252	95% CI P-Value	*NLRC5* rs43216	
		GG	AG+AA
	CC+CT	1.00	0.94 (0.77-1.17) (0.6)
	TT	3.30 (1.39-7.82) **(0.007)**	0.74 (0.48-1.15) (0.18)

Overall p-value: **0.011**

NLRC5 rs27194	95% CI P-Value	*NLRC5* rs289748	
		CC	CT+TT
	AA+AT	1.00	1.05 (0.84-1.32) (0.68)
	TT	3.23 (1.35-7.76) **(0.009)**	0.84 (0.50-1.44) (0.53)

Overall p-value: **0.041**

PD-L1 rs10815225	95% CI P-Value	*NLRC5* rs289726	
		CC	CT+TT
	GG+GC	1.00	0.99 (0.81-1.21) (0.94)
	CC	0.05 (0.007-0.32) **(0.0018)**	1.40 (0.27-7.15) (0.69)

Overall p-value: **0.011**

PD-L1 rs4143815	95% CI P-Value	*NLRC5* rs3751710		
		CC	CT	TT
	GG+GC	1.00	0.82 (0.65-1.04) (0.1)	1.44 (0.81-2.56) (0.22)
	CC	0.68 (0.47-0.99) **(0.044)**	1.67 (0.85-3.30) (0.14)	0.61 (0.16-2.32) (0.47)

Overall p-value: **0.042**

S5 Table. cont.

	95% CI P-Value	*PD-L1* rs4143815	
		GG+GC	CC
PD-L1 rs10815225	GG+GC	1.00	0.87 (0.63-1.20) (0.40)
	CC	0.63 (0.19-2.13) (0.46)	0.016 (0.001-0.17) **(0.0007)**

Overall p-value: **0.009**

	95% CI P-Value	*NLRC5* rs56315364		
		CC	CT	TT
NLRC5 rs27194	AA+AT	1.00	0.96 (0.77-1.21) (0.74)	1.19 (0.89-1.61) (0.25)
	TT	2.02 (0.97-4.20) (0.06)	0.75 (0.41-1.39) (0.36)	6.20 (1.00-38.56) (0.051)

Overall p-value: **0.037**

	95% CI P-Value	*NLRC5* rs1684575		
		GG	GC	CC
PD-L1 rs10815225	TT+GT	1.00	0.97 (0.78-1.21) (0.79)	1.19 (0.89-1.60) (0.23)
	GG	0.11 (0.02-0.62) **(0.013)**	2.27 (0.26-20.10) (0.46)	0.08 (0.006-1.19) (0.067)

Overall p-value: **0.029**

	95% CI P-Value	*PD-L1* rs4143815	
		GG	GC+CC
NLRC5 rs27194	AA+AT	1.00	1.08 (0.88-1.32) (0.46)
	TT	2.68 (1.29-5.57) **(0.008)**	0.89 (0.51-1.54) (0.68)

Overall p-value: **0.046**

	95% CI P-Value	*PD-L1* rs866066	
		CC+CT	TT
NLRC5 rs158483	CC + CT	1.00	0.85 (0.66-1.08) (0.19)
	TT	1.05 (0.71-1.55) (0.80)	2.98 (1.16-7.63) **(0.023)**

Overall p-value: **0.045**

	95% CI P-Value	*NLRC5* rs56315364	
		CC+CT	TT
NLRC5 rs289748	CC+CT	1.00	17.09 (1.28-227.6) (**0.031**)
	TT	0.79 (0.55-1.15) (0.22)	1.22 (0.93-1.59) (0.15)

Overall p-value: **0.016**
