# Supplementary material for: Investigation of single and synergic effects of NLRC5 and PD-L1 variants on the risk of colorectal cancer
Source: PLoS One. 2018 Feb 6;13(2):e0192385. doi: 10.1371/journal.pone.0192385 (PMC5800657; doi:10.1371/journal.pone.0192385)
Supplement: S6 Table — For the best model of each pair, age and sex adjusted ORs and 95% CI, with overall p-values based on the likelihood ratio test, were calculated. (PDF) [file pone.0192385.s006.pdf]

S6 Table. *IFNGR1/2*, *PD-L1* and *NLRC5* pair-wise interactions. For the best model of each pair, age and sex adjusted ORs and 95% CI, with overall p-values based on the likelihood ratio test, were calculated.

IFNGR1 rs2234711	95% CI P-Value	*NLRC5* rs43216	
		GG	AG+AA
	TT	1.00	0.80 (0.52 -1.25) (0.64)
	TC	1.76 (1.12-2.77) **(0.013)**	1.12 (0.74-1.69) (0.59)
	CC	0.90 (0.48-1.69) (0.74)	1.70 (1.00-2.87) (**0.05**)

Overall p-value: **0.002**

IFNGR1 rs2234711	95% CI P-Value	*PD-L1* rs4143815		
		GG	GC	CC
	TT	1.00	1.68 (1.07 - 2.65) **0.025**	0.64 (0.29 - 1.41) 0.27
	TC+CC	2.12 (1.45 - 3.12) **0.0001**	1.65 (1.12 - 2.43) **0.01**	1.67 (0.96 - 2.92) 0.07

Overall p-value: **0.001**

IFNGR2 rs1059293	95% CI P-Value	*NLRC5* rs289747		
		GG	GA	AA
	0	1.00	2.49 (1.59 - 3.91) **<.0001**	1.19 (0.66 - 2.13) 0.56
	1	1.55 (1.15 - 2.07) **0.004**	1.56 (1.08 - 2.26) **0.018**	1.50 (0.96 - 2.35) 0.08
	2	2.39 (1.33 - 4.30) **0.004**	0.98 (0.61 - 1.56) 0.92	1.89 (0.96 - 3.73) 0.07

Overall p-value: **0.001**

IFNGR2 rs1059293	95% CI P-Value	*NLRC5* rs43216		
		GG	GA	AA
	CC	1.00	1.50 (0.92 - 2.44) 0.11	0.76 (0.37 - 1.58) 0.47
	CT+TT	1.49 (0.98 - 2.28) 0.06	0.96 (0.64 - 1.45) 0.85	1.25 (0.74 - 2.1) 0.40

Overall p-value: **0.04**

IFNGR1 rs17181457	95% CI P-Value	*NLRC5* rs56315364	
		CC+CT	TT
	CC	1.00	1.25 (0.87-1.80) (0.23)
	CT+TT	1.25 (0.85-1.83) (0.27)	4.38 (1.72-11.12) **(0.002)**

Overall p-value: **0.006**

IFNGR2 rs17882748	95% CI P-Value	*NLRC5* rs43216		
		GG	GA	AA
	TT+TC	1.00	0.71 (0.52 - 0.97) **0.03**	0.92 (0.59 - 1.43) 0.70
	CC	0.62 (0.39 - 0.98) **0.04**	0.88 (0.57 - 1.37) 0.58	0.31 (0.14 - 0.68) **0.004**

Overall p-value: **0.019**
